# An Evaluative Review of Barriers to Critical Thinking in Educational and Real-World Settings

**DOI:** 10.3390/jintelligence11060105

**Published:** 2023-05-31

**Authors:** Christopher P. Dwyer

**Affiliations:** Technology Education Research Group (TERG), Department of Teacher Education, Athlone Campus, Technological University of the Shannon: Midlands Midwest, N37 HD68 Westmeath, Ireland; christopher.dwyer@tus.ie

**Keywords:** critical thinking, reflective judgment, epistemology, emotion, bias

## Abstract

Though a wide array of definitions and conceptualisations of critical thinking have been offered in the past, further elaboration on some concepts is required, particularly with respect to various factors that may impede an individual’s application of critical thinking, such as in the case of reflective judgment. These barriers include varying levels of epistemological engagement or understanding, issues pertaining to heuristic-based thinking and intuitive judgment, as well as emotional and biased thinking. The aim of this review is to discuss such barriers and evaluate their impact on critical thinking in light of perspectives from research in an effort to reinforce the ‘completeness’ of extant critical thinking frameworks and to enhance the potential benefits of implementation in real-world settings. Recommendations and implications for overcoming such barriers are also discussed and evaluated.

## 1. Introduction

Critical thinking (CT) is a metacognitive process—consisting of a number of skills and dispositions—that, through purposeful, self-regulatory reflective judgment, increases the chances of producing a logical solution to a problem or a valid conclusion to an argument (Dwyer 2017, 2020; Dwyer et al. 2012, 2014, 2015, 2016; Dwyer and Walsh 2019; Quinn et al. 2020). 

CT has long been identified as a desired outcome of education (Bezanilla et al. 2019; Butler et al. 2012; Dwyer 2017; Ennis 2018), given that it facilitates a more complex understanding of information (Dwyer et al. 2012; Halpern 2014), better judgment and decision-making (Gambrill 2006) and less dependence on cognitive bias and heuristic thinking (Facione and Facione 2001; McGuinness 2013). A vast body of research (e.g., Dwyer et al. 2012; Gadzella 1996; Hitchcock 2004; Reed and Kromrey 2001; Rimiene 2002; Solon 2007), including various meta-analyses (e.g., Abrami et al. 2008, 2015; Niu et al. 2013; Ortiz 2007), indicates that CT can be enhanced through targeted, explicit instruction. Though CT can be taught in domain-specific areas, its domain-generality means that it can be taught across disciplines and in relation to real-world scenarios (Dwyer 2011, 2017; Dwyer and Eigenauer 2017; Dwyer et al. 2015; Gabennesch 2006; Halpern 2014). Indeed, the positive outcomes associated with CT transcend educational settings into real-world, everyday situations, which is important because CT is necessary for a variety of social and interpersonal contexts where good decision-making and problem-solving are needed on a daily basis (Ku 2009). However, regardless of domain-specificity or domain-generality of instruction, the transferability of CT application has been an issue in CT research (e.g., see Dumitru 2012). This is an important consideration because issues with transferability—for example, in real-world settings—may imply something lacking in CT instruction.

In light of the large, aforementioned body of research focusing on enhancing CT through instruction, a growing body of research has also evaluated the manner in which CT instruction is delivered (e.g., Abrami et al. 2008, 2015; Ahern et al. 2019; Cáceres et al. 2020; Byerly 2019; Dwyer and Eigenauer 2017), along with additional considerations for and the barriers to such education, faced by teachers and students alike (e.g., Aliakbari and Sadeghdaghighi 2013; Cáceres et al. 2020; Cornell et al. 2011; Lloyd and Bahr 2010; Ma and Liu 2022; Ma and Luo 2021; Rear 2019; Saleh 2019); for example, those regarding conceptualisation, beliefs about CT, having feasible time for CT application and CT’s aforementioned transferability. However, there is a significant lack of research investigating barriers to CT application by individuals in real-world settings, even by those who have enjoyed benefits from previous CT instruction. Thus, perhaps the previously conjectured ‘something lacking in CT instruction’ refers to, in conjunction with the teaching of what CT consists of, making clear to students what barriers to CT application we face.

Simply, CT instruction is designed in such a way as to enhance the likelihood of positive decision-making outcomes. However, there are a variety of barriers that can impede an individual’s application of CT, regardless of past instruction with respect to ‘how to conduct CT’. For example, an individual might be regarded as a ‘critical thinker’ because they apply it in a vast majority of appropriate scenarios, but that does not ensure that they apply CT in *all* such appropriate scenarios. What keeps them from applying CT in those scenarios might well be one of a number of barriers to CT that often go unaddressed in CT instruction, particularly if such instruction is exclusively focused on skills and dispositions. Perhaps too much focus is placed on what educators are teaching their students *to do* in their CT courses as opposed to what educators should be recommending their students to look out for or advising what they *should not* be doing. That is, perhaps just as important for understanding what CT is and how it is conducted (i.e., knowing what to do) is a genuine awareness of the various factors and processes that can *impede* CT; and so, for an individual to think critically, they must know what to look out for and be able to monitor for such barriers to CT application.

To clarify, thought has not changed regarding what CT is or the cognitive/metacognitive processes at its foundation (e.g., see Dwyer 2017; Dwyer et al. 2014; Ennis 1987, 1996, 1998; Facione 1990; Halpern 2014; Paul 1993; Paul and Elder 2008); rather, additional consideration of issues that have potential to negatively impact CT is required, such as those pertaining to epistemological engagement; intuitive judgment; as well as emotional and biased thinking. This notion has been made clear through what might be perceived of as a ‘loud shout’ for CT over at least the past 10–15 years in light of growing political, economic, social, and health-related concerns (e.g., ‘fake news’, gaps between political views in the general population, various social movements and the COVID-19 pandemic). Indeed, there is a dearth of research on *barriers* to CT (Haynes et al. 2016; Lloyd and Bahr 2010; Mangena and Chabeli 2005; Rowe et al. 2015). As a result, this evaluative perspective review aims to provide an impetus for updating the manner in which CT education is approached and, perhaps most importantly, applied in real-world settings—through further identifying and elaborating on specific barriers of concern in order to reinforce the ‘completeness’ of extant CT frameworks and to enhance the potential benefits of their implementation^1^.

## 2. Barriers to Critical Thinking

### 2.1. Inadequate Skills and Dispositions

In order to better understand the various barriers to CT that will be discussed, the manner in which CT is conceptualised must first be revisited. Though debate over its definition and what components are necessary to think critically has existed over the 80-plus years since the term’s coining (i.e., Glaser 1941), it is generally accepted that CT consists of two main components: skills and dispositions (Dwyer 2017; Dwyer et al. 2012, 2014; Ennis 1996, 1998; Facione 1990; Facione et al. 2002; Halpern 2014; Ku and Ho 2010a; Perkins and Ritchhart 2004; Quinn et al. 2020). CT skills—analysis, evaluation, and inference—refer to the higher-order, cognitive, ‘task-based’ processes necessary to conduct CT (e.g., see Dwyer et al. 2014; Facione 1990). CT dispositions have been described as inclinations, tendencies, or willingness to perform a given thinking skill (e.g., see Dwyer et al. 2016; Siegel 1999; Valenzuela et al. 2011), which may relate to attitudinal and intellectual habits of thinking, as well as motivational processes (Ennis 1996; Norris 1994; Paul and Elder 2008; Perkins et al. 1993; Valenzuela et al. 2011). The relationship between CT skills and dispositions has been argued to be mutually dependent. As a result, overemphasising or encouraging the development of one over the other is a barrier to CT as a whole. Though this may seem obvious, it remains the case that CT instruction often places added emphasis on skills simply because they can be taught (though that does not ensure that everyone has or will be taught such skills), whereas dispositions are ‘trickier’ (e.g., see Dwyer 2017; Ku and Ho 2010a). That is, it is unlikely that simply ‘teaching’ students to be motivated towards CT or to value it over short-instructional periods will actually meaningfully enhance it. Moreover, debate exists over how best to train disposition or even measure it. With that, some individuals might be more ‘inherently’ disposed to CT in light of their truth-seeking, open-minded, or inquisitive natures (Facione and Facione 1992; Quinn et al. 2020). The barrier, in this context, is how we can enhance the disposition of those who are not ‘inherently’ inclined. For example, though an individual may possess the requisite skills to conduct CT, it does not ensure the tendency or willingness to apply them; and conversely, having the disposition to apply CT does not mean that one has the ability to do so (Valenzuela et al. 2011). Given the pertinence of CT skills and dispositions to the application of CT in a broader sense, inadequacies in either create a barrier to application.

### 2.2. Epistemological (Mis)Understanding

To reiterate, most extant conceptualisations of CT focus on the tandem working of skills and dispositions, though significantly fewer emphasise the *reflective judgment* aspect of CT that might govern various associated processes (Dawson 2008; Dwyer 2017; Dwyer et al. 2014, 2015; King and Kitchener 1994, 2004; Stanovich and Stanovich 2010). Reflective judgment (RJ) refers to a self-regulatory process of decision-making, with respect to taking time to engage one’s understanding of the nature, limits, and certainty of knowing and how this can affect the defense of their reasoning (Dwyer 2017; King and Kitchener 1994; Ku and Ho 2010b). The ability to metacognitively ‘think about thinking’ (Flavell 1976; Ku and Ho 2010b) in the application of critical thinking skills implies a reflective sensibility consistent with epistemological understanding and the capacity for reflective judgement (Dwyer et al. 2015; King and Kitchener 1994). Acknowledging levels of (un)certainty is important in CT because the information a person is presented with (along with that person’s pre-existing knowledge) often provides only a limited source of information from which to draw a conclusion. Thus, RJ is considered a component of CT (Baril et al. 1998; Dwyer et al. 2015; Huffman et al. 1991) because it allows one to acknowledge that epistemological understanding is necessary for recognising and judging a situation in which CT may be required (King and Kitchener 1994). For example, the interdependence between RJ and CT can be seen in the way that RJ influences the manner in which CT skills like analysis and evaluation are conducted or the balance and perspective within the subsequent inferences drawn (Dwyer et al. 2015; King et al. 1990). Moreover, research suggests that RJ development is not a simple function of age or time but more so a function of the amount of active engagement an individual has working in problem spaces that require CT (Brabeck 1981; Dawson 2008; Dwyer et al. 2015). The more developed one’s RJ, the better able one is to present “a more complex and effective form of justification, providing more inclusive and better integrated assumptions for evaluating and defending a point of view” (King and Kitchener 1994, p. 13).

Despite a lesser focus on RJ, research indicates a positive relationship between it and CT (Baril et al. 1998; Brabeck 1981; Dawson 2008; Dwyer et al. 2015; Huffman et al. 1991; King et al. 1990)—the understanding of which is pertinent to better understanding the foundation to CT barriers. For example, when considering one’s proficiency in CT skills, there might come a time when the individual becomes so good at using them that their application becomes something akin to ‘second nature’ or even ‘automatic’. However, this creates a contradiction: automatic thinking is largely the antithesis of reflective judgment (even though judgment is never fully intuitive or reflective; see Cader et al. 2005; Dunwoody et al. 2000; Hamm 1988; Hammond 1981, 1996, 2000)—those who think critically take their time and reflect on their decision-making; even if the solution/conclusion drawn from the automatic thinking is ‘correct’ or yields a positive outcome, it is not a critically thought out answer, per se. Thus, no matter how skilled one is at applying CT skills, once the application becomes primarily ‘automatic’, the thinking ceases to be *critical* (Dwyer 2017)—a perspective consistent with Dual Process Theory (e.g., Stanovich and West 2000). Indeed, RJ acts as System 2 thinking (Stanovich and West 2000): it is slow, careful, conscious, and consistent (Kahneman 2011; Hamm 1988); it is associated with high cognitive control, attention, awareness, concentration, and complex computation (Cader et al. 2005; Kahneman 2011; Hamm 1988); and accounts for epistemological concerns—consistent not only with King and Kitchener’s (1994) conceptualisation but also Kuhn’s (1999, 2000) perspective on metacognition and *epistemological knowing*. This is where RJ comes into play as an important component of CT—interdependent among the requisite skills and dispositions (Baril et al. 1998; Dwyer et al. 2015)—it allows one to acknowledge that epistemological understanding is vital to recognising and judging a situation in which CT is required (King and Kitchener 1994). With respect to the importance of epistemological understanding, consider the following examples for elaboration.

The primary goal of CT is to enhance the likelihood of generating reasonable conclusions and/or solutions. Truth-seeking is a CT disposition fundamental to the attainment of this goal (Dwyer et al. 2016; Facione 1990; Facione and Facione 1992) because if we just applied any old nonsense as justification for our arguments or solutions, they would fail in the application and yield undesirable consequences. Despite what may seem like truth-seeking’s obvious importance in this context, all thinkers succumb to unwarranted assumptions on occasion (i.e., beliefs presumed to be true without adequate justification). It may also seem obvious, in context, that it is important to be able to distinguish facts from beliefs. However, the concepts of ‘fact’ or ‘truth’, with respect to how much empirical support they have to validate them, also require consideration. For example, some might conceptualise truth as factual information or information that has been or can be ‘proven’ true. Likewise, ‘proof’ is often described as evidence establishing a fact or the truth of a statement—indicating a level of absolutism. However, the reality is that we cannot ‘prove’ things—as scientists and researchers well know—we can only disprove them, such as in experimental settings where we observe a significant difference between groups on some measure—we do not prove the hypothesis correct, rather, we disprove the null hypothesis. This is why, in large part, researchers and scientists use cautious language in reporting their results. We know the best our findings can do is reinforce a theory—another concept often misconstrued in the wider population as something like a hypothesis, as opposed to what it actually entails: a robust model for how and/or why a given phenomenon might occur (e.g., gravity). Thus, theories will hold ‘true’ until they are falsified—that is, disproven (e.g., Popper [1934] 1959, 1999).

Unfortunately, ‘proof’, ‘prove’, and ‘proven’—words that ensure certainty to large populations—actually disservice the public in subtle ways that can hinder CT. For example, a company that produces toothpaste might claim its product to be ‘clinically proven’ to whiten teeth. Consumers purchasing that toothpaste are likely to expect to have whiter teeth after use. However, what happens—as often may be the case—if it does not whiten their teeth? The word ‘proven’ implies a false claim in context. Of course, those in research understand that the word’s use is a marketing ploy, given that ‘clinically proven’ sounds more reassuring to consumers than ‘there is evidence to suggest…’; but, by incorrectly using words like ‘proven’ in our daily language, we reinforce a misunderstanding of what it means to assess, measure and evaluate—particularly from a scientific standpoint (e.g., again, see Popper [1934] 1959, 1999).

Though this example may seem like a semantic issue, it has great implications for CT in the population. For example, a vast majority of us grew up being taught the ‘factual’ information that there were nine planets in our solar system; then, in 2006, Pluto was reclassified as a dwarf planet—no longer being considered a ‘major’ planet of our solar system. As a result, we now have eight planets. This change might be perceived in two distinct ways: (1) ‘science is amazing because it’s always developing—we’ve now reached a stage where we know so much about the solar system that we can differentiate celestial bodies to the extent of distinguishing planets from dwarf planets’; and (2) ‘I don’t understand why these scientists even have jobs, they can’t even count planets’. The first perspective is consistent with that of an individual with epistemological understanding and engagement that previous understandings of models and theories can change, not necessarily because they were wrong, but rather because they have been advanced in light of gaining further credible evidence. The second perspective is consistent with that of someone who has failed to engage epistemological understanding, who does not necessarily see that the change might reflect progress, who might be resistant to change, and who might grow in distrust of science and research in light of these changes. The latter point is of great concern in the CT research community because the unwarranted cynicism and distrust of science and research, in context, may simply reflect a lack of epistemological understanding or engagement (e.g., to some extent consistent with the manner in which conspiracy theories are developed, rationalised and maintained (e.g., Swami and Furnham 2014)). Notably, this should also be of great concern to education departments around the world, as well as society, more broadly speaking.

Upon considering epistemological engagement in more practical, day-to-day scenarios (or perhaps a lack thereof), we begin to see the need for CT in everyday 21st-century life—heightened by the ‘new knowledge economy’, which has resulted in exponential increases in the amount of information made available since the late 1990s (e.g., Darling-Hammond 2008; Dwyer 2017; Jukes and McCain 2002; Varian and Lyman 2003). Though increased amounts of and enhanced access to information are largely good things, what is alarming about this is how much of it is misinformation or disinformation (Commission on Fake News and the Teaching of Critical Literacy in Schools 2018). Truth be told, the new knowledge economy is anything but ‘new’ anymore. Perhaps, over the past 10–15 years, there has been an increase in the need for CT above and beyond that seen in the ‘economy’s’ wake—or maybe ever before; for example, in light of the social media boom, political unrest, ‘fake news’, and issues regarding health literacy. The ‘new’ knowledge economy has made it so that knowledge acquisition, on its own, is no longer sufficient for learning—individuals must be able to work with and adapt information through CT in order to apply it appropriately (Dwyer 2017).

Though extant research has addressed the importance of epistemological understanding for CT (e.g., Dwyer et al. 2014), it does not address how *not* engaging it can substantially hinder it—regardless of how skilled or disposed to think critically an individual may be. Notably, this is distinct from ‘inadequacies’ in, say, memory, comprehension, or other ‘lower-order’ cognitively-associated skills required for CT (Dwyer et al. 2014; Halpern 2014; see, again, Note 1) in that reflective judgment is essentially a pole on a *cognitive continuum* (e.g., see Cader et al. 2005; Hamm 1988; Hammond 1981, 1996, 2000). Cognitive Continuum Theory postulates a continuum of cognitive processes anchored by reflective judgment and intuitive judgment, which represents how judgment situations or tasks relate to cognition, given that thinking is never purely reflective, nor is it completely intuitive; rather, it rests somewhere in between (Cader et al. 2005; Dunwoody et al. 2000). It is also worth noting that, in Cognitive Continuum Theory, neither reflective nor intuitive judgment is assumed, a priori, to be superior (Dunwoody et al. 2000), despite most contemporary research on judgment and decision-making focusing on the strengths of RJ and limitations associated with intuitive judgment (Cabantous et al. 2010; Dhami and Thomson 2012; Gilovich et al. 2002). Though this point regarding superiority is acknowledged and respected (particularly in non-CT cases where it is advantageous to utilise intuitive judgment), in the context of CT, it is rejected in light of the example above regarding the automaticity of thinking skills.

### 2.3. Intuitive Judgment

The manner in which human beings think and the evolution of which, over millions of years, is a truly amazing thing. Such evolution has made it so that we can observe a particular event and make complex computations regarding predictions, interpretations, and reactions in less than a second (e.g., Teichert et al. 2014). Unfortunately, we have become so good at it that we often over-rely on ‘fast’ thinking and intuitive judgments that we have become ‘cognitively lazy’, given the speed at which we can make decisions with little energy (Kahneman 2011; Simon 1957). In the context of CT, this ‘lazy’ thinking is an impediment (as in opposition to reflective judgment). For example, consider a time in which you have been presented numeric data on a topic, and you instantly aligned your perspective with what the ‘numbers indicate’. Of course, numbers do not lie… but people do—that is not to say that the person who initially interpreted and then presented you with those numbers is trying to disinform you; rather, the numbers presented might not tell the full story (i.e., the data are incomplete or inadequate, unbeknownst to the person reporting on them); and thus, there might be alternative interpretations to the data in question. With that, there most certainly are individuals who will wish to persuade you to align with their perspective, which only strengthens the impetus for being aware of intuitive judgment as a barrier. Consider another example: *have you ever accidentally insulted someone at work, school, or in a social setting? Was it because the statement you made was based on some kind of assumption or stereotype?* It may have been an honest mistake, but if a statement is made based on what one thinks they know, as opposed to what they actually know about the situation—without taking the time to recognise that all situations are unique and that reflection is likely warranted in light of such uncertainty—then it is likely that the schema-based ‘intuitive judgment’ is what is a fault here.

Our ability to construct schemas (i.e., mental frameworks for how we interpret the world) is evolutionarily adaptive in that these scripts allow us to: make quick decisions when necessary and without much effort, such as in moments of impending danger, answer questions in conversation; interpret social situations; or try to stave off cognitive load or decision fatigue (Baumeister 2003; Sweller 2010; Vohs et al. 2014). To reiterate, research in the field of higher-order thinking often focuses on the failings of intuitive judgment (Dwyer 2017; Hamm 1988) as being limited, misapplied, and, sometimes, yielding grossly incorrect responses—thus, leading to faulty reasoning and judgment as a result of systematic biases and errors (Gilovich et al. 2002; Kahneman 2011; Kahneman et al. 1982; Slovic et al. 1977; Tversky and Kahneman 1974; in terms of schematic thinking (Leventhal 1984), system 1 thinking (Stanovich and West 2000; Kahneman 2011), miserly thinking (Stanovich 2018) or even heuristics (Kahneman and Frederick 2002; Tversky and Kahneman 1974). Nevertheless, it remains that such protocols are learned—not just through experience (as discussed below), but often through more ‘academic’ means. For example, consider again the anecdote above about learning to apply CT skills so well that it becomes like ‘second nature’. Such skills become a part of an individual’s ‘mindware’ (Clark 2001; Stanovich 2018; Stanovich et al. 2016) and, in essence, become heuristics themselves. Though their application requires RJ for them to be CT, it does not mean that the responses yielded will be incorrect.

Moreover, despite the descriptions above, it would be incorrect, and a disservice to readers to imply that RJ is always right and intuitive judgment is always wrong, especially without consideration of the contextual issues—both intuitive and reflective judgments have the potential to be ‘correct’ or ‘incorrect’ with respect to validity, reasonableness or appropriateness. However, it must also be acknowledged that there is a cognitive ‘miserliness’ to depending on intuitive judgment, in which case, the ability to detect and override this dependence (Stanovich 2018)—consistent with RJ, is of utmost importance if we care about our decision-making. That is, if we *care* about our CT (see below for a more detailed discussion), we must ignore the implicit ‘noise’ associated with the intuitive judgment (regardless of whether or not it is ‘correct’) and, instead, apply the necessary RJ to ensure, as best we can, that the conclusion or solution is valid, reasonable or appropriate.

Although, such a recommendation is much easier said than done. One problem with relying on mental shortcuts afforded by intuition and heuristics is that they are largely experience-based protocols. Though that may sound like a positive thing, using ‘experience’ to draw a conclusion in a task that requires CT is erroneous because it essentially acts as ‘research’ based on a sample size of one; and so, ‘findings’ (i.e., one’s conclusion) cannot be generalised to the larger population—in this case, other contexts or problem-spaces (Dwyer 2017). Despite this, we often over-emphasise the importance of experience in two related ways. First, people have a tendency to confuse experience for expertise (e.g., see the *Dunning–KrugerEffect* (i.e., the tendency for low-skilled individuals to overestimate their ability in tasks relevant to said skill and highly skilled individuals to underestimate their ability in tasks relevant to said skills); see also: (Kruger and Dunning 1999; Mahmood 2016), wherein people may not necessarily be expert, rather they may just have a lot of experience completing a task imperfectly or wrong (Dwyer and Walsh 2019; Hammond 1996; Kahneman 2011). Second, depending on the nature of the topic or problem, people often evaluate experience on par with research evidence (in terms of credibility), given its personalised nature, which is reinforced by self-serving bias(es).

When evaluating topics in domains wherein one lacks expertise, the need for intellectual integrity and humility (Paul and Elder 2008) in their RJ is increased so that the individual may assess what knowledge is required to make a critically considered judgment. However, this is not necessarily a common response to a lack of relevant knowledge, given that when individuals are tasked with decision-making regarding a topic in which they do not possess relevant knowledge, these individuals will generally rely on emotional cues to inform their decision-making (e.g., Kahneman and Frederick 2002). Concerns here are not necessarily about the lack of domain-specific knowledge necessary to make an accurate decision, but rather the (1) belief of the individual that they have the knowledge necessary to make a critically thought-out judgment, even when this is not the case—again, akin to the Dunning–Kruger Effect (Kruger and Dunning 1999); or (2) lack of willingness (i.e., disposition) to gain additional, relevant topic knowledge.

One final problem with relying on experience for important decisions, as alluded to above, is that when experience is engaged, it is not necessarily an objective recollection of the procedure. It can be accompanied by the individual’s beliefs, attitudes, and feelings—how that experience is recalled. The manner in which an individual draws on their personal experience, in light of these other factors, is inherently emotion-based and, likewise, biased (e.g., Croskerry et al. 2013; Loftus 2017; Paul 1993).

### 2.4. Bias and Emotion

Definitions of CT often reflect that it is to be applied to a topic, argument, or problem of importance that the individual *cares* about (Dwyer 2017). The issue of ‘caring’ is important because it excludes judgment and decision-making in day-to-day scenarios that are *not* of great importance and *do not* warrant CT (e.g., ‘what colour pants best match my shirt’ and ‘what to eat for dinner’); again, for example, in an effort to conserve time and cognitive resources (e.g., Baumeister 2003; Sweller 2010). However, given that ‘importance’ is subjective, it essentially boils down to what one *cares* about (e.g., issues potentially impactful in one’s personal life; topics of personal importance to the individual; or even problems faced by an individual’s social group or work organisation (in which case, care might be more extrinsically-oriented). This is arguably one of the most difficult issues to resolve in CT application, given its contradictory nature—where it is generally recommended that CT should be conducted void of emotion and bias (as much as it can be possible), at the same time, it is also recommended that it should only be applied to things we care about. As a result, the manner in which care is conceptualised requires consideration. For example, in terms of CT, care can be conceptualised as ‘concern or interest; the attachment of importance to a person, place, object or concept; and serious attention or consideration applied to doing something correctly or to avoid damage or risk’; as opposed to some form of *passion* (e.g., *intense, driving or over-powering feeling or conviction; emotions as distinguished from reason; a strong liking or desire for or devotion* to some activity, object or concept). In this light, care could be argued as more of a dispositional or self-regulatory factor than emotional bias; thus, making it useful to CT. Though this distinction is important, the manner in which care is labeled does not lessen the potential for biased emotion to play a role in the thinking process. For example, it has been argued that if one cares about the decision they make or the conclusion they draw, then the individual will do their best to be objective as possible (Dwyer 2017). However, it must also be acknowledged that this may not always be the case or even completely feasible (i.e., *how can any decision be fully void of emotional input?*)—though one may strive to be as objective as possible, such objectivity is not ensured given that *implicit* bias may infiltrate their decision-making (e.g., taking assumptions for granted as facts in filling gaps (unknowns) in a given problem-space). Consequently, such implicit biases may be difficult to amend, given that we may not be fully aware of them at play.

With that, explicit biases are just as concerning, despite our awareness of them. For example, the more important an opinion or belief is to an individual, the greater the resistance to changing their mind about it (Rowe et al. 2015), even in light of evidence indicating the contrary (Tavris and Aronson 2007). In some cases, the provision of information that corrects the flawed concept may even ‘backfire’ and reinforce the flawed or debunked stance (Cook and Lewandowsky 2011). This cognitive resistance is an important barrier to CT to consider for obvious reasons—as a process; it acts in direct opposition to RJ, the skill of evaluation, as well as a number of requisite dispositions towards CT, including truth-seeking and open-mindedness (e.g., Dwyer et al. 2014, 2016; Facione 1990); and at the same time, yields important real-world impacts (e.g., see Nyhan et al. 2014).

The notion of emotion impacting rational thought is by no means a novel concept. A large body of research indicates a negative impact of emotion on decision-making (e.g., Kahneman and Frederick 2002; Slovic et al. 2002; Strack et al. 1988), higher-order cognition (Anticevic et al. 2011; Chuah et al. 2010; Denkova et al. 2010; Dolcos and McCarthy 2006) and cognition, more generally (Iordan et al. 2013; Johnson et al. 2005; Most et al. 2005; Shackman et al. 2006)^2^. However, less attention has specifically focused on emotion’s impact on the application of *critical* thought. This may be a result of assumptions that if a person is inclined to think critically, then what is yielded will typically be void of emotion—which is true to a certain extent. However, despite the domain generality of CT (Dwyer 2011, 2017; Dwyer and Eigenauer 2017; Dwyer et al. 2015; Gabennesch 2006; Halpern 2014), the likelihood of emotional control during the CT process remains heavily dependent on the topic of application. Consider again, for example; there is no guarantee that an individual who generally applies CT to important topics or situations will do so in *all* contexts. Indeed, depending on the nature of the topic or the problem faced, an individual’s mindware (Clark 2001; Stanovich 2018; Stanovich et al. 2016; consistent with the metacognitive nature of CT) and the extent to which a context can evoke emotion in the thinker will influence what and how thinking is applied. As addressed above, if the topic is something to which the individual feels passionate, then it will more likely be a greater challenge for them to remain unbiased and develop a reasonably objective argument or solution.

Notably, self-regulation is an important aspect of both RJ and CT (Dwyer 2017; Dwyer et al. 2014), and, in this context, it is difficult not to consider the role *emotional intelligence* might play in the relationship between affect and CT. For example, though there are a variety of conceptualisations of emotional intelligence (e.g., Bar-On 2006; Feyerherm and Rice 2002; Goleman 1995; Salovey and Mayer 1990; Schutte et al. 1998), the underlying thread among these is that, similar to the concept of self-regulation, emotional intelligence (EI) refers to the ability to monitor (e.g., perceive, understand and regulate) one’s own feelings, as well as those of others, and to use this information to guide relevant thinking and behaviour. Indeed, extant research indicates that there is a positive association between EI and CT (e.g., Afshar and Rahimi 2014; Akbari-Lakeh et al. 2018; Ghanizadeh and Moafian 2011; Kaya et al. 2017; Stedman and Andenoro 2007; Yao et al. 2018). To shed light upon this relationship, Elder (1997) addressed the potential link between CT and EI through her description of the latter as a measure of the extent to which affective responses are *rationally-based*, in which *reasonable* desires and behaviours emerge from such rationally-based emotions. Though there is extant research on the links between CT and EI, it is recommended that future research further elaborate on this relationship, as well as with other self-regulatory processes, in an effort to further establish the potentially important role that EI might play within CT.

## 3. Discussion

### 3.1. Interpretations

Given difficulties in the past regarding the conceptualisation of CT (Dwyer et al. 2014), efforts have been made to be as specific and comprehensive as possible when discussing CT in the literature to ensure clarity and accuracy. However, it has been argued that such efforts have actually added to the complexity of CT’s conceptualisation and had the opposite effect on clarity and, perhaps, more importantly, the accessibility and practical usefulness for educators (and students) not working in the research area. As a result, when asked what CT is, I generally follow up the ‘long definition’, in light of past research, with a much simpler description: CT is akin to ‘playing devil’s advocate’. That is, once a claim is made, one should second-guess it in as many conceivable ways as possible, in a process similar to the Socratic Method. Through asking ‘why’ and conjecturing alternatives, we ask the individual—be it another person or even ourselves—to justify the decision-making. It keeps the thinker ‘honest’, which is particularly useful if we’re questioning ourselves. If we do not have justifiable reason(s) for why we think or intend to act in a particular way (above and beyond considered objections), then it should become obvious that we either missed something or we are biased. It is perhaps this simplified description of CT that gives such impetus for the aim of this review.

Whereas extant frameworks often discuss the importance of CT skills, dispositions, and, to a lesser extent, RJ and other self-regulatory functions of CT, they do so with respect to components of CT or processes that facilitate CT (e.g., motivation, executive functions, and dispositions), without fully encapsulating cognitive processes and other factors that may hinder it (e.g., emotion, bias, intuitive judgment and a lack of epistemological understanding or engagement). With that, this review is neither a criticism of existing CT frameworks nor is it to imply that CT has so many barriers that it cannot be taught well, nor does it claim to be a complete list of processes that can impede CT (see again Note 1). To reiterate, education in CT can yield beneficial effects (Abrami et al. 2008, 2015; Dwyer 2017; Dwyer and Eigenauer 2017); however, such efficacy may be further enhanced by presenting students and individuals interested in CT the barriers they are likely to face in its application; explaining how these barriers manifest and operate; and offer potential strategies for overcoming them.

### 3.2. Further Implications and Future Research

Though the barriers addressed here are by no means new to the arena of research in higher-order cognition, there is a novelty in their collated discussion as impactful barriers in the context of CT, particularly with respect to extant CT research typically focusing on introducing strategies and skills for enhancing CT, rather than identifying ‘preventative measures’ for barriers that can negatively impact CT. Nevertheless, future research is necessary to address how such barriers can be overcome in the context of CT. As addressed above, it is recommended that CT education include discussion of these barriers and encourage self-regulation against them; and, given the vast body of CT research focusing on enhancement through training and education, it seems obvious to make such a recommendation in this context. However, it is also recognised that simply identifying these barriers and encouraging people to engage in RJ and self-regulation to combat them may not suffice. For example, educators might very well succeed in teaching students how to apply CT *skills*, but just as these educators may not be able to motivate students to use them as often as they might be needed or even to value such skills (such as in attempting to elicit a positive disposition towards CT), it might be the case that without knowing about the impact of the discussed barriers to CT (e.g., emotion and/or intuitive judgment), students may be just as susceptible to biases in their attempts to think critically as others without CT skills. Thus, what such individuals might be applying is not CT at all; rather, just a series of higher-order cognitive skills from a biased or emotion-driven perspective. As a result, a genuine understanding of these barriers is necessary for individuals to appropriately self-regulate their thinking.

Moreover, though the issues of epistemological beliefs, bias, emotion, and intuitive processes are distinct in the manner in which they can impact CT, these do not have set boundaries; thus, an important implication is that they can overlap. For example, epistemological understanding can influence how individuals make decisions in real-world scenarios, such as through intuiting a judgment in social situations (i.e., without considering the nature of the knowledge behind the decision, the manner in which such knowledge interacts [e.g., correlation v. causation], the level of uncertainty regarding both the decision-maker’s personal stance and the available evidence), when a situation might actually require further consideration or even the honest response of ‘I don’t know’. The latter concept—that of simply responding ‘I don’t know’ is interesting to consider because though it seems, on the surface, to be inconsistent with CT and its outcomes, it is commensurate with many of its associated components (e.g., intellectual honesty and humility; see Paul and Elder 2008). In the context this example is used, ‘I don’t know’ refers to epistemological understanding. With that, it may also be impacted by bias and emotion. For example, depending on the topic, an individual may be likely to respond ‘I don’t know’ when they do not have the relevant knowledge or evidence to provide a sufficient answer. However, in the event that the topic is something the individual is emotionally invested in or feels passionate about, an opinion or belief may be shared instead of ‘I don’t know’ (e.g., Kahneman and Frederick 2002), despite a lack of requisite evidence-based knowledge (e.g., Kruger and Dunning 1999). An emotional response based on belief may be motivated in the sense that the individual knows that they do not know for sure and simply uses a belief to support their reasoning as a persuasive tool. On the other hand, the emotional response based on belief might be used simply because the individual may not know that the use of a belief is an insufficient means of supporting their perspective– instead, they might think that their intuitive, belief-based judgment is as good as a piece of empirical evidence; thus, suggesting a lack of empirical understanding. With that, it is fair to say that though epistemological understanding, intuitive judgment, emotion, and bias are distinct concepts, they can influence each other in real-world CT and decision-making. Though there are many more examples of how this might occur, the one presented may further support the recommendation that education can be used to overcome some of the negative effects associated with the barriers presented.

For example, in Ireland, students are not generally taught about academic referencing until they reach third-level education. Anecdotally, I was taught about referencing at age 12 and had to use it all the way through high school when I was growing up in New York. In the context of these referencing lessons, we were taught about the credibility of sources, as well as how analyse and evaluate arguments and subsequently infer conclusions in light of these sources (i.e., CT skills). We were motivated by our teacher to find the ‘truth’ as best we could (i.e., a fundament of CT disposition). Now, I recognise that this experience cannot be generalised to larger populations, given that I am a sample size of one, but I do look upon such education, perhaps, as a kind of transformative learning experience (Casey 2018; King 2009; Mezirow 1978, 1990) in the sense that such education might have provided a basis for both CT and epistemological understanding. For CT, we use research to support our positions, hence the importance of referencing. When a ‘reference’ is not available, one must ask if there is actual evidence available to support the proposition. If there is not, one must question the basis for why they think or believe that their stance is correct—that is, where there is logic to the reasoning or if the proposition is simply an emotion- or bias-based intuitive judgment. So, in addition to referencing, the teaching of some form of epistemology—perhaps early in children’s secondary school careers, might benefit students in future efforts to overcome some barriers to CT. Likewise, presenting examples of the observable impact that bias, emotions, and intuitive thought can have on their thinking might also facilitate overcoming these barriers.

As addressed above, it is acknowledged that we may not be able to ‘teach’ people not to be biased or emotionally driven in their thinking because it occurs naturally (Kahneman 2011)—regardless of how ‘skilled’ one might be in CT. For example, though research suggests that components of CT, such as disposition, can improve over relatively short periods of time (e.g., over the duration of a semester-long course; Rimiene 2002), less is known about *how* such components have been enhanced (given the difficulty often associated with trying to *teach* something like disposition (Dwyer 2017); i.e., to reiterate, it is unlikely that simply ‘teaching’ (or telling) students to be motivated towards CT or to value it (or its associated concepts) will actually enhance it over short periods of time (e.g., semester-long training). Nevertheless, it is reasonable to suggest that, in light of such research, educators can encourage dispositional growth and provide opportunities to develop it. Likewise, it is recommended that educators encourage students to be aware of the cognitive barriers discussed and provide chances to engage in CT scenarios where such barriers are likely to play a role, thus, giving students opportunities to acknowledge the barriers and practice overcoming them. Moreover, making students aware of such barriers at younger ages—in a simplified manner, may promote the development of personal perspectives and approaches that are better able to overcome the discussed barriers to CT. This perspective is consistent with research on RJ (Dwyer et al. 2015), in which it was recommended that such enhancement requires not only time to develop (be it over the course of a semester or longer) but is also a function of having increased opportunities to engage CT. In the possibilities described, individuals may learn both to overcome barriers to CT and from the positive outcomes of applying CT; and, perhaps, engage in some form of transformative learning (Casey 2018; King 2009; Mezirow 1978, 1990) that facilitates an enhanced ‘valuing’ of and motivation towards CT. For example, through growing an understanding of the nature of epistemology, intuitive-based thinking, emotion, bias, and the manner in which people often succumb to faulty reasoning in light of these, individuals may come to better understand the limits of knowledge, barriers to CT and how both understandings can be applied; thus, growing further appreciation of the process as it is needed.

To reiterate, research suggests that there may be a developmental trajectory above and beyond the parameters of a semester-long training course that is necessary to develop the RJ necessary to think critically and, likewise, engage an adequate epistemological stance and self-regulate against impeding cognitive processes (Dwyer et al. 2015). Though such research suggests that such development may not be an issue of time, but rather the amount of opportunities to engage RJ and CT, there is a dearth of recommendations offered with respect to how this could be performed in practice. Moreover, the *how* and *what* regarding ‘opportunities for engagement’ requires further investigation as well. For example, does this require additional academic work outside the classroom in a formal manner, or does it require informal ‘exploration’ of the world of information on one’s own? If the latter, the case of motivational and dispositional levels once again comes into question; thus, even further consideration is needed. One way or another, future research efforts are necessary to identify how best to make individuals aware of barriers to CT, encourage them to self-regulate against them, and identify means of increasing opportunities to engage RJ and CT.

## 4. Conclusions

Taking heed that it is unnecessary to reinvent the CT wheel (Eigenauer 2017), the aim of this review was to further elaborate on the processes associated with CT and make a valuable contribution to its literature with respect to conceptualisation—not just in light of making people explicitly aware of what it is, but also what it is not and how it can be impeded (e.g., through inadequate CT skills and dispositions; epistemological misunderstanding; intuitive judgment; as well as bias and emotion)—a perspective consistent with that of ‘constructive feedback’ wherein students need to know both what they are doing right and what they are doing wrong. This review further contributes to the CT education literature by identifying the importance of (1) engaging understanding of the nature, limits, and certainty of knowing as individuals traverse the landscape of evidence-bases in their research and ‘truth-seeking’; (2) understanding how emotions and biases can affect CT, regardless of the topic; (3) managing gut-level intuition until RJ has been appropriately engaged; and (4) the manner in which language is used to convey meaning to important and/or abstract concepts (e.g., ‘caring’, ‘proof’, causation/correlation, etc.). Consistent with the perspectives on research advancement presented in this review, it is acknowledged that the issues addressed here may not be complete and may themselves be advanced upon and updated in time; thus, future research is recommended and welcomed to improve and further establish our working conceptualisation of critical thinking, particularly in a real-world application.

## Data Availability

No new data were created or analyzed in this study. Data sharing is not applicable to this article.

## Notes

1 Notably, though inadequacies in cognitive resources (apart from those explicitly set within the conceptualisations of CT discussed; e.g., see Section 2.1) are acknowledged as impediments to one’s ability to apply CT (e.g., a lack of relevant background knowledge, as well as broader cognitive abilities and resources (Dwyer 2017; Halpern 2014; Stanovich and Stanovich 2010)), these will not be discussed as focus is largely restricted to issues of cognitive processes that ‘naturally’ act as barriers in their functioning. Moreover, such inadequacies may more so be issues of individual differences than ongoing issues that *everyone*, regardless of ability, would face in CT (e.g., the impact of emotion and bias). Nevertheless, it is recommended that future research further investigates the influence of such inadequacies in cognitive resources on CT.

2 There is also some research that suggests that emotion may mediate enhanced cognition (Dolcos et al. 2011, 2012). However, this discrepancy in findings may result from the types of emotion studied—such as task-relevant emotion and task-irrelevant emotion. The distinction between the two is important to consider in terms of, for example, the distinction between one’s general mood and feelings specific unto the topic under consideration. Though mood may play a role in the manner in which CT is conducted (e.g., making judgments about a topic one is passionate about may elicit positive or negative emotions that affect the thinker’s mood in some way), notably, this discussion focuses on task-relevant emotion and associated biases that negatively impact the CT process. This is also an important distinction because an individual may generally think critically about ‘important’ topics, but may fail to do so when faced with a cognitive task that requires CT with which the individual has a strong, emotional perspective (e.g., in terms of *passion*, as described above).
